# A concise review of chewing gum as an anti-cariogenic agent

**DOI:** 10.3389/froh.2023.1213523

**Published:** 2023-06-13

**Authors:** Clara Yan-Yu Yeung, Chun-Hung Chu, Ollie Yiru Yu

**Affiliations:** Faculty of Dentistry, The University of Hong Kong, Hong Kong, Hong Kong SAR, China

**Keywords:** chewing gum, sugar-free gum, dental caries, caries management, cariology, preventive dentistry, oral health

## Abstract

Chewing gum has been endorsed as a caries preventive agent by the FDI World Dental Federation, the American Dental Association, and the European Food Safety Authority. This review discusses the mechanism and provides an update of the use of chewing gum for caries prevention. Chewing gum typically consists of a water-insoluble gum base, water-soluble added ingredients, and active ingredients. It can be classified as sugar-containing or sugar-free, as well as nonmedicated or medicated. Chewing gum prevents dental caries through a range of mechanisms, including the clearance of the oral cavity, neutralization of oral acidity, inhibition of cariogenic bacterial growth, remineralization of enamel, and reduction of appetite. Recent clinical studies have evaluated the efficacy of sugar-free chewing gum for caries prevention, with most demonstrating positive results, although some studies have reported contradictory outcomes. To achieve optimal caries prevention, it is generally recommended that individuals chew sugar-free gum for five minutes after meals, three times daily.

## 1. Introduction

Dental caries is one the most prevalent health conditions worldwide. Half of the world's population is affected by untreated caries. It can cause pain and infections and undermines one's general health and quality of life (1). Treating dental caries can be expensive. The World Health Organization (WHO) reported that 5%–10% of healthcare budgets were used for the treatment of dental caries in industrialized countries (2). Therefore, effective and cost-effective preventive strategies for caries prevention are essential and beneficial to the population and the health care system.

Chewing gum can be used for caries prevention with economic benefits to the healthcare sector (3–5). Chewing gum is a sweetened and flavoured insoluble plastic material used for chewing. Humans have a long history of using chewing gum-like agents. Chewing betel was found in Asia and Oceania 4,000 years ago, and chewing coca leaf was found in ancient Andes 3,000 years ago (6, 7). Despite thousands of years of history, modern chewing gum was developed and commercialized in 1,848 (8).

Chewing gum contains a water-insoluble gum base, water-soluble added ingredients, and active ingredients (9, 10). The water-insoluble gum base is composed of elastomers, elastomer solvents, and fillers. They are nonnutritive and cannot be dissolved during the chewing process. Water-soluble added ingredients include bulking agents, flavouring agents, antioxidants, sweeteners, colourants, opacifiers, softeners, emulsifiers, anti-tack agents, and anti-caking agents. They optimize the physical and chemical properties of chewing gum (11). Active ingredients can also be added to the chewing gum for therapeutic use. With the addition of active ingredients, some chewing gum can be used for medical purpose, such as nicotine-containing chewing gum for smoke cessation or aspirin-containing chewing gum for pain relief (9, 10). Because this review focused on the role of chewing gum as a caries preventive agent, medicated chewing gum, which does not have the caries preventive function, is not discussed in this review. Examples of the active ingredient of chewing gum for dental use includes casein phosphopeptide–amorphous calcium phosphate-nanocomplexes (CPP-ACP), fluoride, carbamide, or chlorhexidine (Table 1) (20).

**Table 1:** Composition of the chewing gum.

Ingredient	Function	Composition	Examples
Water-insoluble gum base			
Elastomer	Provides elasticity and cohesiveness increase flexibility against breaking or cracking (9)	15%–45%	Chicle, nispero, polyisobutylene, isobutyleneisoprene copolymers
Elastomer solvents	Bind the elastomers and the fillers by softening elastomeric materials (9)	15%	Glycerol esters, terpene resins
Fillers	Provide overall texture and facilitates blending during the processing stages; alter the chewing ability of the gum (9)	50%	Magnesium, calcium carbonate, ground limestone
Water-soluble added ingredients			
Bulking agents	Produce required bulk for drug incorporation (9, 10)	Varies	Polydextrose, insulin
Flavoring agents	Improve flavor (9)	0.01%–1%	Natural and artificial volatile essential oils, synthetic flavours
Antioxidants	Protect gum base and flavors from oxidation, prevent growth of microorganisms (9, 12)	0.02%	Propyl gallate, butylated hydroxy anisole
Sweeteners	Provide sweet taste of the gum, Polyols have low rate of metabolism and acid production by oral bacteria, increase net plaque pH	Varies	Caloric sweeteners: sugars (monosaccharide, disaccharide) Non-caloric sweeteners: sugar alcohols/polyols (sorbitol, mannitol, xylitol, maltitol or the blend of these); artificial sweeteners: aspartame, saccharin and acesulfame K (9)
Colorants	Provide and improve color (9, 13)	0.1%	FD&C-approved colors (fruit and vegetable extracts)
Opacifiers	Provide whiteness (9)	0.5%–2%	Titanium dioxide, magnesium oxide
Softeners	Provide softness by regulate cohesiveness and modify texture, Create better mouth feel (9, 13)	0.5%–15%	Glycerin, fatty acids.
Emulsifiers	Improve softness and provide the hydration effect (9, 10)	15%–45%	Mono-, di-, tri-, stearyl acetate and lactylic esters
Anti-tack agents	Reduce fragmentation of gum during mastication, prevent gum attaches to teeth or denture (14)	0.2%–0.6%	α-cellulose, vegetable proteins
Anti-caking agents	Prevent caking and forming lumps; improve flowability, rehydration and facilitate packaging; extent shelf life (9, 13)	0.5%–2%	Precipitated silicon dioxide, solid carbon dioxide.
Active ingredients			
Calcium and phosphate salts	Supplement natural calcium and phosphate levels of saliva, promote remineralization (15)	Varies	Casein phosphopeptide–amorphous calcium phosphate complexes (CPP-ACP)
Urea (carbamide)	Facilitate the neutralization of plaque pH (16)	Varies	N/A
Fluorides	Enhance remineralization, inhibit microbial growth and metabolism (17)	Varies	N/A
Antimicrobials	Reduce dental plaque formation (18, 19)	Varies	Natural antimicrobials (magnolia bark extract) Synthetic antimicrobials (chlorhexidine and triclosan)

Chewing gum has been recognized as a caries preventive agent by several professional organizations, including the European Food Safety Authority since 2010, FDI world dental association since 2015, and the American Dental Association since 2021 (21). Although two recent systematic reviews reported the caries preventive effect of sugar-free chewing gum and xylitol-containing sugar-free chewing gum, respectively (21, 22), the evidence and knowledge of chewing gum in caries prevention has not been comprehensively updated (21). Therefore, this review article aims to provide an overview and update on chewing gum as a caries preventive agent.

## 2. Methods

### 2.1. Data sources and selection

The literature search was conducted in three databases, including PubMed/Medline, Web of Science, and Scopus to identify the available studies evaluating the prevention of caries lesions with chewing gum.

### 2.2. Search strategy

The search strategy was developed as follows:

1. “demineralization” OR “tooth demineralization” OR “teeth demineralization” OR “caries” OR “carious” OR “tooth decay” OR “teeth decay” OR “dental caries” OR “caries susceptibility”
2. “chewing gum” OR “sugar-free gum”
3. “[1]” AND “[2]”

### 2.3. Inclusion criteria

**Figure 1:**
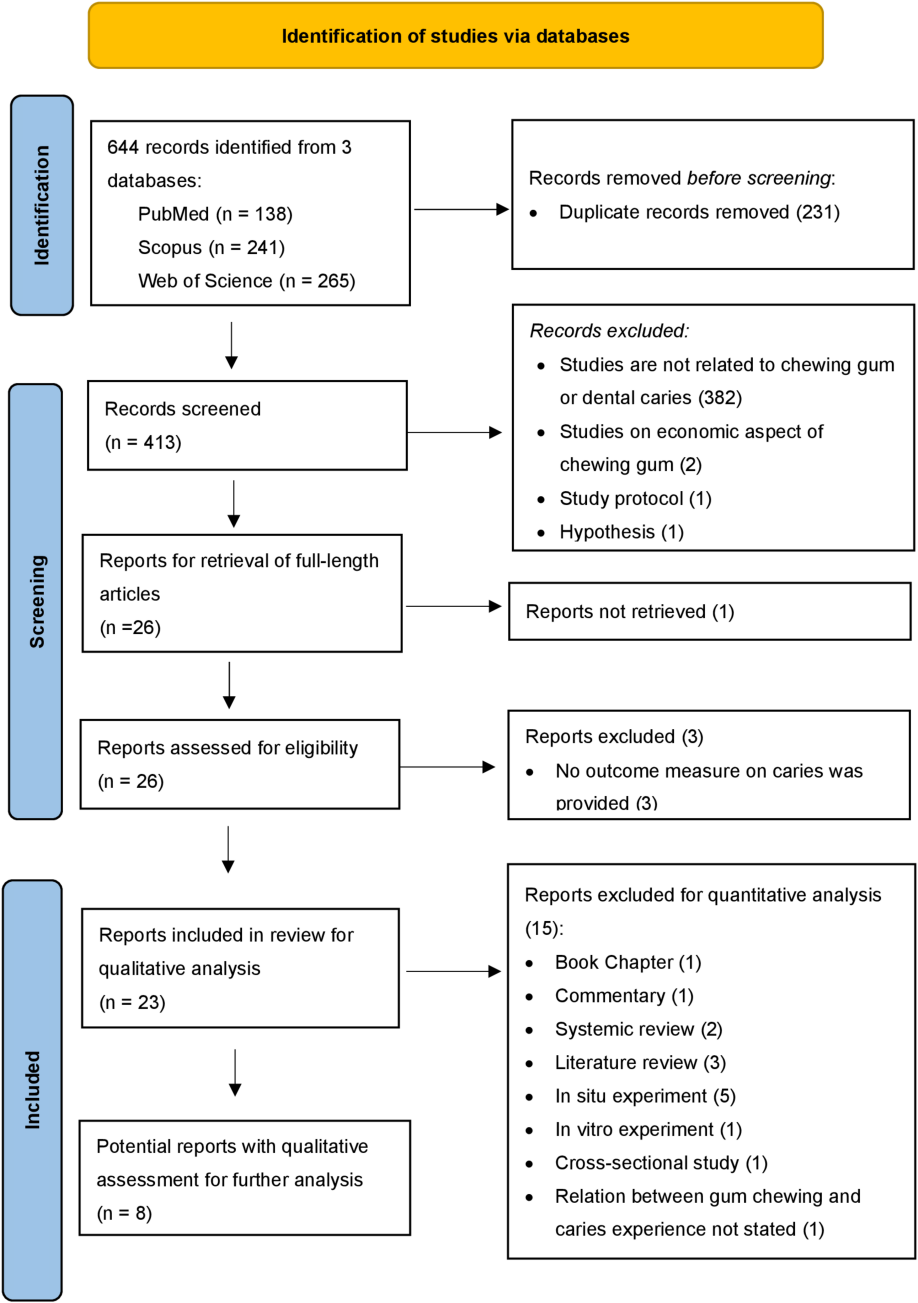
Flow chart of study identification, screening, and inclusion.

English articles on the caries preventive effect of chewing gum in children or adults published from 2012 to 2022 were included. Randomized controlled trials, cross-sectional studies, pre-post trials, and any clinical studies designed with a comparative arm were identified and analysed for eligibility of further quantitative analysis (Figure 1).

### 2.4. Data collection and analysis

The data on the clinical performance of chewing gum in caries prevention were extracted and analysed.

## 3. Classification

### 3.1. Classification by sugar contents

Based on the sugar content, chewing gum can be classified as sugar-containing chewing gum or sugar-free chewing gum. Sugar-containing chewing gum is chewing gum that contains simple carbohydrates, such as monosaccharides and disaccharides, in which monosaccharides include glucose, fructose, and galactose, and disaccharides include maltose, sucrose, and lactose. Chewing gum containing any of these are considered sugar-containing chewing gum (9, 10, 12, 20). Sugar-free chewing gum does not contain simple carbohydrates. It can be further classified into chewing gum containing sugar alcohol and chewing gum containing other sweeteners. Sugar alcohol includes polyols, such as xylitol, sorbitol, etc. Non sugar alcohol-containing gum is sugar-free chewing gum. They might contain other sweeteners, such as artificial sweeteners like aspartame and saccharin. Non sugar alcohol-containing gum can also be sweetener-free, which is plain chewing gum (9, 10, 12, 20).

### 3.2. Classification by active ingredients

Chewing gum can be classified by its active ingredients. Based on whether the chewing gum contains bioactive drug content or not, chewing gum can be classified as medicated chewing or nonmedicated chewing gum. Medicated chewing gum contains active ingredients. Examples include CPP-ACP-containing chewing gum, carbamide-containing chewing gum, fluoride-containing chewing gum, and chlorhexidine-containing gum (9, 10, 12, 20).

## 4. Effectiveness in caries prevention

Conducting a meta-synthesis was not feasible in the current review due to the high heterogeneity among studies. The included clinical studies on the caries preventive effect of chewing gum used various outcomes to assess the caries experience of the participants, including increments of decayed, missing, filled teeth (DMFT), increments of decayed, missing, filled surfaces (DMFS), increments of decayed surfaces (DS), increments of International Caries Detection and Assessment System (ICDAS) coding, and volume change of demineralized lesions. Therefore, the details of the included clinical studies were presented without further processing of the data (Table 2). The results of the included studies were summarized as follows.

**Table 2:** Randomized clinical trials of chewing gum published from 2012 to 2022.

Study	Intervention (Duration)	Chewing time (Frequency)	Participants (age)	Follow up	Outcome measure	Result (*p*-value)	*p*
Al-Haboubi et al. 2012 (23)	Gp1: Xylitol Gp2: No gum (6 m)	15 min, (2x/day)	146 adults (> 60 y)	6 m	Increments of DMFS	Gp1: 3.1 Gp2: 2.9	0.627
Alamoudi et al. 2012 (24)	Gp1: Xylitol Gp2: Fluoride varnish (3 m)	5 min/x, (3x/d)	34 mothers (22–45 y)	18 m	Increments of DMFT	Gp1: −0.2 Gp2: 4.91	0.001
Campus et al. 2013 (25)	Gp1: Xylitol Gp2: Placebo gum (6 m)	5 min/x, (5x/d)	148 children (7–9 y)	2 y	Increments of decayed tooth surfaces	Enamel Lesion: Gp1: 0.13 Gp2: 0.67 Dentine lesion: Gp1: 0.01 Gp2: 0.18	< 0.05
Tao et al. 2013 (26)	Gp1: Tea polyphenol + Xylitol Gp2: Xylitol Gp3: No gum (2y)	8 min/x, (2x/d)	157 children (8–9 y)	2 y	Increments of DMFT	Gp1: 0.17 Gp2: 0.60 Gp3: 1.15	<0.001
Dong et al. 2014 (27)	Gp1: 3 g Xylitol Gp2: 5 g Xylitol Gp3: No gum (12 w)	Gp 1: 20 min/x, (3x/d) Gp 2: 12 min/x, (5x/d) Gp 3 (N.A)	155 students (8–13 y)	12 w	Decrease of volume of the demineralized lesion	Gp1: 8.48 Gp2: 12.09 Gp3: 1.56	< 0.05
Cocco et al. 2017 (28)	Gp1: Xylitol Gp2: Placebo gum (1 y)	5 min/x, (3x/d)	130 adults (30–45 y)	2 y	Increments of ICDAS	Initial Lesion: Gp1: 0.14 Gp2: 0.20	0.01
** **						Moderate Lesion: Gp1: 0.16 Gp2: 0.18	0.12
** **						Extensive Lesion: Gp1: 0.30 Gp2: 0.44	0.03
Watthanasaen et al. 2017 (29)	Gp1: Xylitol Gp2: No gum **(**1 y**)**	5 min/x, (3x/d)	174 students (7–18 y)	1 y	Increments of ICDAS on primary teeth	Gp1: 2.83 Gp2: 3.62	< 0.05
** **	** **				Increments of ICDAS on permanent teeth	Gp1: 15.25 Gp2: 16.31	< 0.05
Cagettia et al. 2020 (30)	Gp1: Magnolia + Xylitol Gp2: Xylitol Gp3: Placebo gum (1 y)	5 min/x, (3x/d)	194 adults (30–45 y)	2 y	Increments of ICDAS	Initial Lesion Gp1: 0.10 Gp2: 0.14 Gp3: 0.20	< 0.01
** **						Moderate Lesion Gp1: 0.14 Gp2: 0.16 Gp3: 0.18	0.10
** **						Extensive Lesion: Gp1: 0.25 Gp2: 0.30 Gp3: 0.44	< 0.01

Gp, group; x, times; min, minutes; d, day; m, month; y, year; RCT, randomized controlled trial; DMFS, decayed/missing/filled surfaces of permanent dentition; DMFT, decayed/missing/filled teeth of permanent dentition; dmft, decayed/missing/filled surfaces of primary dentition; ICDAS, international caries detection and assessment system; Enamel lesion, clinically detectable enamel lesion without cavitation and cavity limited to enamel; Dentine lesion, cavity involving dentine; placebo gum, polyol-containing gum; Tea polyphenol + Xylitol, Tea polyphenol and xylitol-containing gum; Magnolia + Xylitol, Magnolia and xylitol-containing gum.

### 4.1. Effects of chewing gum compared to no gum

Four studies investigated the caries preventive effect of chewing gum compared to no gum (23, 26, 27, 29). Three out of four studies showed that chewing gum lowered caries increments in comparison to no gum use, with prevention ranging from 6.95% to 85.2% (26, 27, 29). One study did not find a caries preventive effect of chewing gum (23).

### 4.2. Effects of chewing gum compared to fluoride varnish

One study investigated the use of chewing gum compared to fluoride varnish (24). It showed chewing gum reduced more caries formation in comparison to fluoride varnish.

### 4.3. Types of chewing gum on caries preventive effects

Three studies investigated the caries preventive effect of xylitol-containing chewing gum compared to polyol-containing chewing gum (25, 28, 30). One study showed that xylitol-containing chewing gum had a better caries preventive effect than other polyol-containing chewing gum in both initial enamel caries and dentine caries (25). Another two studies were conducted with the same clinical trial (28, 30). They showed xylitol-containing chewing gum had a better caries preventive effect for initial and extensive lesions as defined in the ICDAS than other polyol-containing chewing gum. However, such an effect was insignificant in moderated lesions. One study investigated the caries preventive effect of tea polyphenol-containing chewing gum compared to xylitol-containing chewing gum (26). The study showed that tea polyphenol-containing chewing gum had a superior caries preventive effect in comparison to xylitol-containing chewing gum. Another study investigated the caries preventive effect of magnolia-containing chewing gum compared to xylitol-containing chewing gum (30). The results indicated that magnolia-containing chewing gum had a superior caries preventive effect in comparison to xylitol-containing chewing gum for initial and extensive lesions but not moderate (30).

### 4.4. Duration of the intervention on caries preventive effects

The caries preventive effect of chewing gum compared to no gum was found in a varied intervention period of daily chewing gum use, ranging from 12 weeks to two years (24, 26, 27, 29). With as short as a 12-week daily use of chewing gum, a reduced caries experience was observed (27).

### 4.5. Frequency and duration of chewing on caries preventive effects

One study compared the caries preventive effects of chewing gum to no gum with two different frequencies and durations (5 times a day and 12 min each time vs. 3 times a day and 20 min each time) yet the same daily total duration of 60 min (27). The group with higher frequency and lower duration of chewing gum daily presented a superior remineralization effect. However, it should also be noted that the total daily intake of xylitol is higher in the former group (5 g) than the latter (3 g).

### 4.6. Effects of chewing gum on different age groups

Three studies investigated the caries preventive effect of chewing gum compared to no gum on children and adolescents (26, 27, 29). All studies showed that chewing gum lowered caries increments in comparison to no gum use, with prevention ranging from 6.95% to 85.2% (26, 27, 29). One study investigated the use of chewing gum on participants above 60 years old and compared to no gum (23). The results did not show chewing gum reduce caries experience on elderly. No included studies investigated the use of chewing gum compared to no gum on adults.

## 5. Mechanism in caries prevention

Sugar-free chewing gum prevents dental caries by clearing the oral cavity, neutralizing the acidity of oral cavity, remineralizing enamel, inhibiting the growth of cariogenic bacteria, and reducing appetite.

### 5.1. Oral cavity clearance

Chewing gum prevents caries by clearing the oral cavity. Two systemic reviews concluded the anti-plaque effect of sugar free chewing gum (31, 32). When debris of food containing fermentable carbohydrates is retained in the oral cavity, it can be fermented by cariogenic bacteria to produce acid (33). Chewing gum can facilitate the mechanical removal of food debris, which contains dietary sugars (34). In addition, chewing gum increases salivary flow rates, which creates an increased flushing effect against food debris. Through stimulating saliva and reducing plaque mechanically, chewing gum can improve oral hygiene (35).

### 5.2. Neutralization of acidity

The acidity of oral cavity will reduce after the oral cavity being exposure to fermentable carbohydrates. When the pH level is below 5.5, the critical pH of enamel, demineralization of the underlying enamel occurs (36). Chewing gum can increase the secretion of saliva. The increased salivary stimulation can last for 9 min with a 187% increase of saliva in the first minute, and a 86% increase in the subsequent minute in comparison to unstimulated status (37). Besides, the buffering capacity of stimulated saliva is higher compared to unstimulated saliva (38, 39). Furthermore, some studies suggested that there will be a slight increase of the unstimulated salivary pH (40). All these facilitate the neutralization of acidity in the oral cavity and reduced enamel demineralization.

### 5.3. Remineralization of enamel

Chewing gum can stimulate the production of saliva, which is supersaturated with calcium and phosphate ions. These ions can precipitate to the demineralized enamel surface and promote remineralization (34, 41).

### 5.4. Inhibition of cariogenic bacteria growth

Previous studies show that xylitol-containing chewing gum can reduce the level of Streptococcus mutans in the oral cavity by inhibiting the attachment of S. mutans to the tooth surface (42, 43). In addition, xylitol cannot be metabolized by S. mutans, but it can compete with mono- and polysaccharides in the metabolic pathways of S. mutans and inhibit the production of lactic acid by the bacteria (44).

### 5.5. Reduction of appetite

Consuming chewing gum after a meal might lower the appetite of the consumer and subsequent snack intake (33). A clinical trial reported that the group chewing gum after lunch experienced a lower level of snack intake, desire for sweet food, and subjective feeling of hunger in comparison to the no-gum group (45). In another trial, the group chewing gum after lunch also experienced a lower level of snack intake, especially carbohydrate-containing food in comparison to the no-gum group (46).

## 6. Application

Despite the anti-caries effect of chewing gum, using chewing gum alone is insufficient to achieve caries prevention. Chewing gum should be used in adjunction with tooth brushing with fluoridated toothpaste twice a day and daily cleaning of interdental area (47).

### 6.1. Daily intake

In order to achieve the caries preventive effects of chewing gum, the amount, duration, and time of intake is important. According to the included clinical studies, a positive caries preventive effect was found with participants chewing gum after a meal at least 3 times per day. Therefore, the best time to chew gum is after a snack or meal at least 3 times per day. The European Food Safety Authority suggests chewing gum for 20 min each time (48). However, the anti-caries effect of chewing gum can be achieved after 5 min of chewing per use according to the included studies (47).

### 6.2. Choice of chewing gum

Sugar-free gum is suggested for caries prevention. Despite multiple clinical studies (49) favouring xylitol-containing gum over sorbitol-containing gum, a systemic review in 2012 (49) suggested that the current evidence supporting xylitol over sorbitol is contradictory. More research is needed on the topic. Although studies showed a superior caries preventive effect of magnolia bark extract-containing gum and tea polyphenol-containing gum in comparison to xylitol-containing gum (20, 25, 30), they are not available commercially. Research on the caries preventive effect of chewing gum containing other active ingredients, such as CPP-ACP, fluoride, and urease, is lacking.

## 7. Potential adverse effects if overuse or abuse

Despite the multiple advantages of chewing gum and the caries prevention effects as stated above, some studies have suggested the potential adverse effects of chewing gum (20), often relating to the excessive chewing process and the excessive consumption of ingredients in chewing gum. The potential adverse effects include choking, jaw muscle pain, temporo-mandibular joint disorder, headache, mercury release from amalgam restoration, and diarrhoea.

### 7.1. Choke

There have been incidents of choking on chewing gum, especially in young children (50, 51). According to the U.S. Department of Agriculture, children under the age of 4 should not chew gum. As for children above that age, the risk of chewing during eating is smaller. Good eating habits are believed to prevent choking (52).

### 7.2. Jaw muscle pain and temporo-mandibular joint disorder (TMD)

There are studies reporting arthralgia and myofascial pain after the excessive chewing of gum for over three hours per day (53, 54). The prolonged exercise of the jaw muscle which exceeds its capacity may lead to temporo-mandibular joint disorder (TMD) and a pain in the jaw muscles (55). It is, therefore, important to avoid prolonged gum chewing.

### 7.3. Headache

Some studies suggested excessive chewing gum use is related to chronic headaches among adolescents (56). The mechanism behind this is still unclear. The chronic headache is hypothesized to be related to the temporo-mandibular joint dysfunction provoked by excessive gum chewing or the consumption of aspartame that chewing gum contains (56, 57).

### 7.4. Mercury release

Chewing gum can accelerate the release of mercury for individuals with dental amalgam fillings. The amount of mercury in plasma and urine for individuals with regular gum chewing habits are significantly higher than those without such habits (58).

### 7.5. Diarrhoea

There are cases reporting diarrhoea and abdominal discomfort after chewing gum consumption (59). This can be related to the over consumption of polyols in gum, which can cause gastrointestinal disturbances and lead to irritable bowel syndrome. This negative effect is dose-dependent (60). The amount of polyol in chewing gum is low and would not cause any gastrointestinal effects in most individuals (60, 61).

## 8. Summary

Chewing gum can be classified as sugar-containing chewing gum or sugar-free chewing gum based on the sugar content or as nonmedicated chewing gum or medicated chewing gum based on the active ingredients. Chewing gum prevents dental caries by clearing the oral cavity, neutralizing the acidity in the oral cavity, inhibiting the growth of cariogenic bacteria, remineralizing enamel, and reducing the appetite. The caries preventive effect of the sugar-free chewing gum has been proved in many clinical studies, though a few included studies showed a contradictory result. A proper duration of chewing gum for 5 min after a meal for 3 times per day is recommended to avoid potential adverse effects.
